# Effectiveness of an ankle–foot orthosis on walking in patients with stroke: a systematic review and meta-analysis

**DOI:** 10.1038/s41598-021-95449-x

**Published:** 2021-08-05

**Authors:** Yoo Jin Choo, Min Cheol Chang

**Affiliations:** 1 Production R&D Division Advanced Interdisciplinary Team, Medical Device Development Center, Daegu-Gyeongbuk Medical Innovation Foundation, Daegu, Republic of Korea; 2 Department of Rehabilitation Medicine, College of Medicine, Yeungnam University, Daegu, Republic of Korea; 3 Department of Physical Medicine and Rehabilitation, College of Medicine, Yeungnam University, 317-1, Daemyungdong, Namku, Daegu, 705-717 Republic of Korea

**Keywords:** Neuroscience, Medical research

## Abstract

We conducted a meta-analysis to investigate the effectiveness of ankle–foot orthosis (AFO) use in improving gait biomechanical parameters such as walking speed, mobility, and kinematics in patients with stroke with gait disturbance. We searched the MEDLINE (Medical Literature Analysis and Retrieval System Online), CINAHL (Cumulative Index to Nursing and Allied Health Literature), Cochrane, Embase, and Scopus databases and retrieved studies published until June 2021. Experimental and prospective studies were included that evaluated biomechanics or kinematic parameters with or without AFO in patients with stroke. We analyzed gait biomechanical parameters, including walking speed, mobility, balance, and kinematic variables, in studies involving patients with and without AFO use. The criteria of the Cochrane Handbook for Systematic Reviews of Interventions were used to evaluate the methodological quality of the studies, and the level of evidence was evaluated using the Research Pyramid model. Funnel plot analysis and Egger’s test were performed to confirm publication bias. A total of 19 studies including 434 participants that reported on the immediate or short-term effectiveness of AFO use were included in the analysis. Significant improvements in walking speed (standardized mean difference [SMD], 0.50; 95% CI 0.34–0.66; P < 0.00001; I^2^, 0%), cadence (SMD, 0.42; 95% CI 0.22–0.62; P < 0.0001; I^2^, 0%), step length (SMD, 0.41; 95% CI 0.18–0.63; P = 0.0003; I^2^, 2%), stride length (SMD, 0.43; 95% CI 0.15–0.71; P = 0.003; I^2^, 7%), Timed up-and-go test (SMD, − 0.30; 95% CI − 0.54 to − 0.07; P = 0.01; I^2^, 0%), functional ambulation category (FAC) score (SMD, 1.61; 95% CI 1.19–2.02; P < 0.00001; I^2^, 0%), ankle sagittal plane angle at initial contact (SMD, 0.66; 95% CI 0.34–0.98; P < 0.0001; I^2^, 0%), and knee sagittal plane angle at toe-off (SMD, 0.39; 95% CI 0.04–0.73; P = 0.03; I^2^, 46%) were observed when the patients wore AFOs. Stride time, body sway, and hip sagittal plane angle at toe-off were not significantly improved (p = 0.74, p = 0.07, p = 0.07, respectively). Among these results, the FAC score showed the most significant improvement, and stride time showed the lowest improvement. AFO improves walking speed, cadence, step length, and stride length, particularly in patients with stroke. AFO is considered beneficial in enhancing gait stability and ambulatory ability.

## Introduction

Stroke is a neurological disease whose sequelae are associated with physical disabilities^1^. Gait limitations are noted in > 50% of patients with stroke, and these limitations may be attributable to motor or proprioceptive impairment, spasticity, and balancing problems^2^. Impaired gait function after stroke strongly contributes to overall patient disability and increases the risk of falls^3^. Weakness in the ankle dorsiflexors is frequently observed after a stroke, which is one of the major factors hindering gait function^4^. Because of ankle dorsiflexor weakness, bodily instability occurs during the stance phase of gait, and the foot is dragged along the ground during the swing phase^5^. With this instability and foot dragging, walking becomes unsafe^5^. In clinical practice, ankle–foot orthoses (AFOs) are recommended for improving the gait limitations of patients. However, some clinicians have reported that AFOs can hinder the natural walking patterns of patients with stroke or hemiplegia^6–8^.

Some previously published systematic reviews or meta-analyses have assessed the effect of AFO on gait function in patients with stroke. In 2013, Tyson et al. found that AFO was effective in improving gait function but only evaluated the kinematics and oxygen consumption^9^. In 2018, Daryabor et al. reported that any type of AFO could improve foot drop but did not proceed with statistical analysis^10^. In 2020, Darybor et al., in a systematic review, reported that AFO could improve walking energy costs in patients with stroke in the short term^11^, and Shahabi et al. reported that AFO could improve walking speed in patients with stroke, but other gait-related factors were not analyzed^12^.

In our meta-analysis for a detailed evaluation of the effectiveness of AFO, we attempted to examine various gait-related variables, including walking speed, cadence, step length, stride length, stride time, Timed up-and-go test (TUG), functional ambulation category (FAC), body sway, ankle sagittal plane angle at initial contact, knee sagittal plane angle at toe-off, and hip sagittal plane angle at toe-off.

## Materials and methods

### Search strategy

In this study, the PICO (population, intervention, comparison, outcome) model for establishing the search strategy was set as follows: (1) population—patients diagnosed with stroke in subacute (1 to 6 months) or chronic stages (more than 6 months)^13^; (2) intervention—walking with an AFO; (3) comparison—walking without an AFO; and (4) outcome—gait parameters (walking speed, cadence, step length, stride length, stride time, ankle sagittal plane angle at initial contact, knee sagittal plane angle at toe-off, hip sagittal plane angle at toe-off), gait ability (FAC score), balance parameter (body sway), and both of gait ability and balance parameter (TUG time). This meta-analysis was conducted according to the Preferred Reporting Items for Systematic Reviews and Meta-analysis (PRISMA) guidelines. We searched trial registers and databases including MEDLINE (Medical Literature Analysis and Retrieval System Online), CINAHL (Cumulative Index to Nursing and Allied Health Literature), Cochrane, Embase, and Scopus for studies published up to June 2021. Articles with insufficient or non-existent gait biomechanical and kinematic variable data, case reports, unpublished papers, and non-English language publications were excluded. The keywords used for searching were as follows: (stroke OR cerebrovascular diseases) AND (orthotic devices OR braces OR splints OR foot OR ankle) (Supplementary 1).

### Inclusion and exclusion criteria

The following studies were included: (1) clinical trials involving patients with stroke, (2) studies comparing the results of evaluations in patients with and without AFO use, (3) studies with passive AFO, (4) studies with an experimental design in which outcomes were measured with all participants in the study with or without an AFO, (5) prospective studies, and (6) studies with full text. Studies involving an AFO with an electrical stimulation function or powered AFO, such as robotic devices, and studies with insufficient results or no data were excluded.

### Data extraction

All search results were exported into the EndNote X9 software tool. After excluding duplicate articles using the deduplication function of EndNote X9, two reviewers independently assessed the potentially eligible studies meeting the selection criteria. The studies were selected by reviewing the titles and abstracts. Subsequently, the qualifications were confirmed through a full-text review of the selected studies. When a disagreement occurred, the decision was determined by consensus between the two reviewers. If more than one study identified the same variable, those studies were included in the meta-analysis. Table 1 shows the information on the number of participants, age, mean time since stroke, types of AFOs used in the experiment, and evaluation tools. All data were presented as mean and standard deviation.

**Table 1:** Characteristics of the selected studies.

No	Study	Subjects	Intervention	Measurement methods and outcomes
1	Abe et al.^14^	N = 16 (mean age = 55.9 ± 11.8 y, mean time since stroke = 31.1 mo) Able to walk at least 8 m, 4 times bare feet without external support except from a cane	With or without plastic ankle–foot orthosis	5-m walk test Stride length, step length, step width, velocity, cadence, step-length symmetry ratio Coefficient of variation of spatial parameters Unaffected-side step length coefficient of variation, step-width coefficient of variation Functional ambulation category
2	Burdett et al.^15^	N = 11 (mean age = 61.9 ± 10.7 y, mean time since stroke = 114.5 ± 108.5 d) Able to ambulate unassisted or with a conventional or quad cane	No orthosis, Air-Stirrup® orthosis (with inflatable air cells), plastic (adjustable plantar flexion/dorsiflexion) and metal (adjustable plantar flexion) ankle–foot orthosis	Videotaped trials, footprint analyses Stride time, stride length, speed, base of support, step length, toe-out angle, hip-knee-ankle sagittal plane angle
3	Chen et al.^16^	N = 24 (mean age = 58.9 ± 9.5 y, mean time since stroke = 13 mo) Able to stand without external support for 60 s and to perform anterior–posterior and lateral weight shifting	With or without anterior ankle–foot orthosis (with anterior leaf spring)	Computer dyno graphy system Static postural stability Postural sway index, body weight on the affected leg Dynamic postural stability Maximal balance range (anterior–posterior, left–right), body weight on the affected leg
4	Corcoran et al.^17^	N = 15 (mean age = 45.1 y, mean time since stroke = 40.3 mo) Able to walk unassisted over a distance of 1000 ft (about 300 m) without stopping	No orthosis, plastic ankle–foot orthosis (with solid anterior closure), standard metal ankle–foot orthosis	Speed-controlled respirometer for ambulation measurement (SCRAM) Walking speed, energy expenditure
5	de Wit et al.^18^	N = 20 (mean age 61.2 y, mean time since stroke = 25.6 mo) Able to walk independently with shoes with and without orthosis	Plastic ankle foot orthosis (with posterior steel), nonarticulated ankle–foot orthosis	10-m walkway Velocity Timed up-and-go test Gait speed Stair test Gait speed Functional ambulation category
6	Dogan et al.^19^	N = 51 (mean age = 60.7 ± 12.5 y, mean time since stroke = 69.2 ± 30.2 d) Able to walk	With or without ankle–foot orthosis (articulated, plantar flexion stopped)	Berg balance scale Timed up-and-go test The stroke rehabilitation assessment of movement measure
7	Farmani et al.^20^	N = 18 (mean age = 57.86 ± 10.44 y, mean time since stroke = 25.31 ± 16 mo) Able to walk independently over at least 10 m without an assistive device	Barefoot, solid ankle–foot orthosis (adjustable plantar flexion/dorsiflexion), rocker bar ankle–foot orthosis	Vicon motion analysis system Gait velocity, cadence, step length, step width, hip extension at toe-off, knee flexion at toe-off, pre-swing time
8	Gatti et al.^21^	N = 10 (mean age = 45.5 y, mean time since stroke = 40 mo) Able to walk at least 10 m without external support	With or without standard plastic ankle–foot orthosis	ELITE (motion capture system) 10-m walk test Knee flexion angle at toe off, peak knee flexion angle, gait speed, step length of the nonparetic limb
9	Gök et al.^22^	N = 12 (mean age = 54 y, mean time since stroke = 67 d)	No orthosis, Seattle-type plastic ankle–foot orthosis (adjustable dorsiflexion), metallic ankle–foot orthosis (adjustable dorsiflexion)	Vicon motion analysis system Cadence, walking speed, single step time, double support time, single step length, ankle dorsiflexion angle in stance/swing phase, knee flexion moment
10	Hesse et al.^23^	N = 19 (mean age = 55.2 y, mean time since stroke = 5.1 mo) Able to walk 20 m barefoot without physical help	Barefoot, Valens caliper (one-bar metal ankle–foot orthosis, with anterior soft closure)	Rivermead Motor Assessment (RMA) 10-m walk test Velocity, cadence, stride length Infotronic force shoe system Stance/swing symmetry, double-stance duration, length of the trajectories of the force point of action
11	Hesse et al.^24^	N = 21 (mean age = 58.2 y, mean time since stroke = 4.9 mo) Able to walk 20 m barefoot without physical help by a therapist	Barefoot, Valens caliper (one-bar metal ankle–foot orthosis, with anterior soft closure)	10-m walk test Gait velocity, cadence, stride length Biaxial goniometers Angle of ankle dorsiflexion, vertical ground reaction forces at heel-on and at toe-off, durations of stance/swing/double support
12	Hung et al.^25^	N = 52 (median age = 54.5 y, median time since stroke = 33.5 mo) Able to walk 10 m with or without an assistive device	With or without plastic anterior ankle–foot orthosis	Modified emory functional ambulation profile Time to ambulate 5-m walk test on a hard floor 5-m walk test on a carpet Timed up-and-go test Standardized obstacle-course Ascending and descending stairs 6-m walk test Walking endurance
13	Pohl et al.^26^	N = 28 (mean age = 51.7 y, mean time since stroke = 2.6 mo) Able both to stand without an assistive device for 20 s and to walk with or without walking aids for 15 m	With or without ankle–foot orthosis (combination of soft-cast/hard-cast material)	ADDON system Postural sway, stance symmetry, gait symmetry parameters vertical/horizontal ground reaction forces, double stance duration
14	Simons et al.^27^	N = 20 (mean age = 57.2 y, mean time since stroke = 39.3 mo) Able to walk over 10 m with or without an assistive device	With or without ankle–foot orthosis - Four types of ankle–foot orthosis (n = 5 each); nonarticulated plastic ankle–foot orthosis with small posterior steel/with two crossed posterior steels and an open heel/large posterior heel, articulated metal ankle–foot orthosis with double bars attached to the outsole of a normal shoe	Caren (computer-controlled 6-degrees-of-freedom motion platform) Static and dynamic weight-bearing asymmetry, dynamic balance contribution Berg Balance Scale Timed up-and-go test 10-m walking test Functional ambulation category Timed balance test Functional balance
15	Tyson et al.^28^	N = 25 (mean age = 49.9 y, mean time since stroke = 8.3mo) Able to bear weight and step with the weak leg	With or without plastic hinged ankle–foot orthosis (adjustable plantar flexion)	5-m walkway Weak/sound stride length, weak/sound step length, step symmetry, cadence, velocity Functional ambulation category
16	Tyson et al.^29^	N = 20 (mean age = 65.6 ± 10.4 y, mean time since stroke = 6.5 wk) Able to walk 5 m without physical support	With or without off-the-shelf ankle–foot orthosis	5-m walk test Walking speed, step length Functional ambulation category
17	Wang et al.^30^	N = 42 (mean age = 59.9 ± 13.0 y, mean time since stroke:101.0 ± 51.3 d) Able to walk over 10 m with or without an assistive device	With or without off-the-shelf ankle–foot orthosis	Balance master system Static balance Weight-bearing difference in standing, body sway Dynamic balance Movement velocity, maximum excursion, directional control Sit to standing Rising time, weight transfer, center of gravity sway 10-m walk test Gait speed, cadence Berg Balance Scale
18	Yamamoto et al.^31^	N = 40 (mean age = 59.9 ± 10.9 y, mean time since stroke: ankle–foot orthosis with plantarflexion stop group = 78.4 ± 47.3 d; ankle–foot orthosis with plantarflexion resistance group = 68.9 ± 24.0 d) Able to walk 10 m without ankle–foot orthosis	With or without metal ankle–foot orthosis Two types of ankle–foot orthosis; metal ankle–foot orthosis with plantarflexion stop using a Klenzak joint, metal ankle–foot orthosis with plantarflexion resistance using an oil damper	Vicon motion analysis system Temporal and distance factors Velocity, paretic to nonparetic, nonparetic to paretic, cycle time, loading response time, single-stance time, pre-swing time, swing time Ground reaction forces Max posterior component, max anterior component Center of pressure progression Progression in loading response, progression in single stance Joint kinematics and kinetics Ankle, knee, and hip joint angles and moments Pelvic and thoracic tilt angles
19	Zollo et al.^32^	N = 10 (mean age = 64.3 ± 10.8 y, mean time since stroke = 64.4 ± 72.84 mo) Able to walk without assistance, with or without support	Solid ankle–foot orthosis (made of plastic, with posterior leaf), dynamic ankle–foot orthosis (made of carbon fiber, with anterior leaf)	Lower-extremity Fugl-Meyer Mini-mental state examination Modified Ashworth scale Passive range of motion Timed up-and-go test Cadence, stride time, step length, stride length, percentage of the swing phase, percentage of the double support phase, ankle/knee/hip range of motion

y, years; mo, months; wk, weeks.

### Quality and level of evidence assessments

The methodological quality was evaluated using the criteria described in the Cochrane Handbook for Systematic Reviews of Interventions to assess the causes of potential bias. The sources of bias included the following: (1) selection bias (random sequence generation, allocation concealment), (2) performance bias (blinding of participants and personnel), (3) detection bias (blinding of outcome assessment), (4) attrition bias (incomplete outcome data), (5) reporting bias (selective reporting), and (6) other bias. Additionally, the level of evidence was defined using the Research Pyramid model: level 1, systematic reviews and meta-analysis of randomized controlled trials; level 2, one or more randomized controlled trials; level 3, controlled trials without randomization; level 4, case–control or cohort study; level 5, systematic review of descriptive and qualitative studies; level 6, single descriptive or qualitative study; and level 7, expert opinion. The results of the level of evidence evaluation of the papers included in this review were level 2 in 15 papers^14,16–18,20–22,25–32^ and level 3 in 4 papers^15,19,23,24^.

### Analyses

A review management software (RevMan 5.3) was used for statistical analysis of the pooled data. For each analysis, a heterogeneity test was performed using I^2^ statistics, which measures the extent of inconsistency among results. When I^2^ values were ≤ 50%, the pooled data were considered homogeneous, and the fixed-effect model was applied. In contrast, if I^2^ values were > 50%, the pooled data were considered to have substantial heterogeneity, and the random-effect model was used for data analyses. The analyzed data were continuous variables; therefore, we calculated the standardized mean differences (SMDs) and 95% confidence intervals (Cis).

P-values < 0.05 were considered to indicate statistical significance. A meta-analysis was performed only when two or more studies could be compared for each survey item. In cases in which two or more orthoses were used, all data on each orthosis used were included in the analysis.

## Results

### Study selection

**Figure 1:**
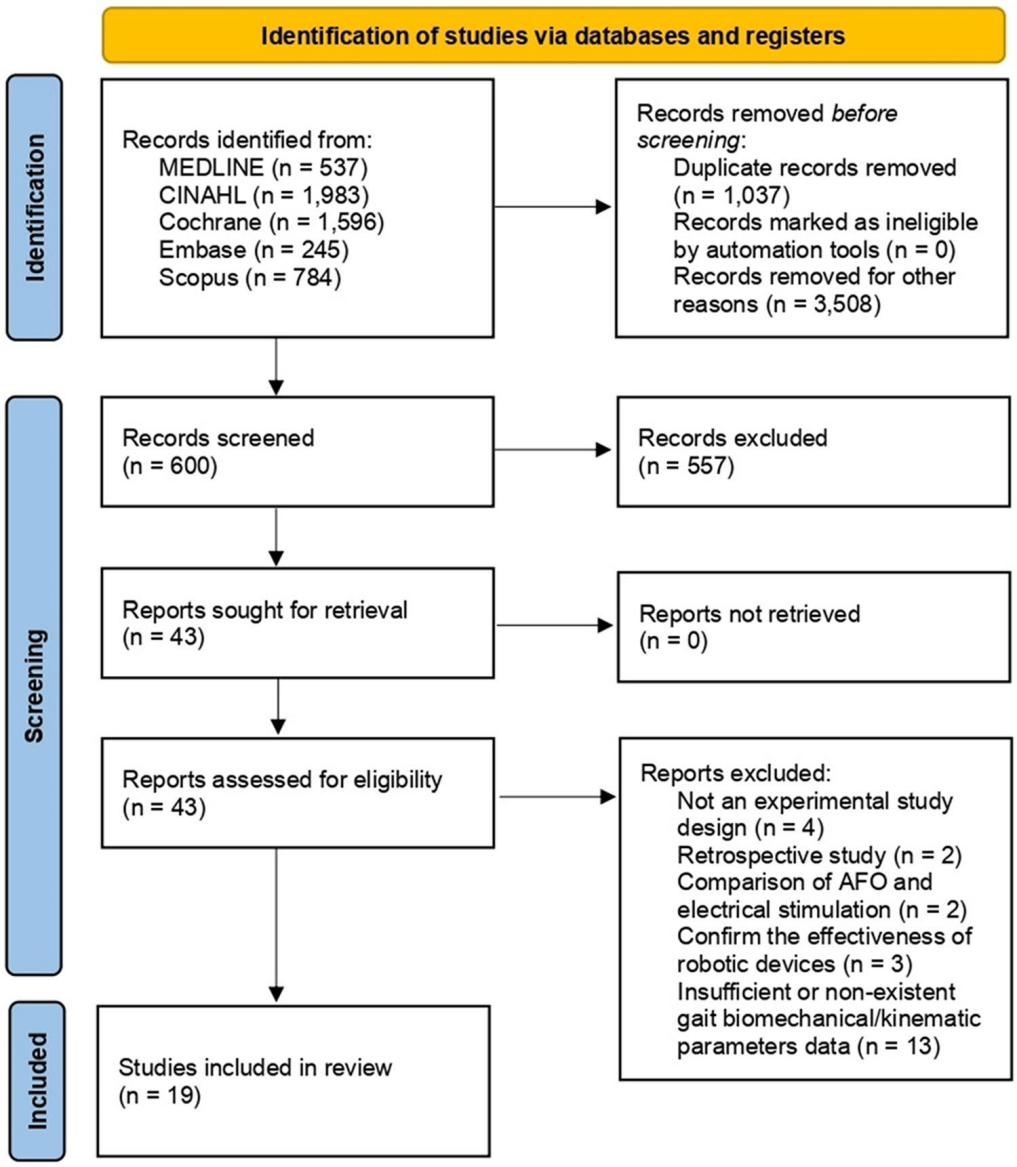
Flowchart showing the search results of the meta-analysis.

From a total of 5145 papers retrieved using the keywords, 43 were selected after excluding duplicate articles or articles discordant with the subject, articles with unclear data, and those whose full contents could not be identified. Among these, we verified the study design, AFO type used, participants’ characteristics, number of participants included, outcome variables (biomechanical and kinematic parameters), and assessment tools. 11 articles were excluded because their study designs and interventions did not meet our criteria, and 13 were excluded owing to insufficient or nonexistent data. Therefore, a total of 19 papers^14–32^ were included in this study (Fig. 1). The number of subjects included in each study was at least 10, and 434 patients were included in the analysis. The average duration of stroke onset of the subjects included in each study was confirmed: 9 studies^15,19,22–24,26,29–31^ included patients with stroke in subacute stages and 10 studies^14,16–18,20,21,25,27,28,32^ included patients with stroke in chronic stages. Each participant could stand independently or walk for at least 8 m without assistive devices or human assistance. There were 9 studies^15,16,18,19,22,25–27,29^ in which subjects had previously worn an AFO or gait assistive device before performing the test. Participants in Burdett et al.’s study^15^ were 11 people who used metal or plastic AFOs and 8 people did not wear AFO before the study proceeded. In the studies by Chen et al.^16^ and Dogan et al.^19^, prior to the start of the study, subjects had experience in using AFO in the post-stroke rehabilitation period, and there was no mention of the duration of use. Subjects included in de Wit et al.’s study^18^ had experience wearing non-articulated plastic AFOs daily for at least 6 months. In the study by Hung et al.^25^, potential participants were required to wear an AFO for at least 5 months prior to the study. Pohl et al.^26^ included subjects who had used foot orthosis for less than 1 week. Simons et al.^27^ recommended that participants use AFO daily for a minimum of months prior to study commencement. In the studies by Chen et al.^16^ and Gök et al.^22^, participants had experience using a cane and the duration of use was not stated. In one of these studies^16^, 19 out of 24 used a regular cane and a 4-quad cane, and in the other^22^, all participants used a single or 3-point cane. In the studies of Gök et al.^22^ and Tyson et al.^29^, before measuring outcomes to confirm the immediate effects of AFO, subjects were given time to practice applying them to AFO. Participants included in the study by Yamamoto et al.^31^ received gait rehabilitation from a physical therapist before participation but did not use AFO. In other studies, there was no mention of the experience of wearing an AFO or gait assistive device. The type of AFO used in the test was different, but the AFO with a built-in electrical stimulation function or robotic device was not used. The intervention groups for all studies included wearing an AFO, and the control group included those who were barefoot or wearing shoes without the AFO. The analysis included articles confirming the immediate (immediately after AFO application) or short-term (1 week to 6 months after AFO application) effectiveness of AFO use. The participants in all studies randomly selected the order of interventions when measuring outcomes.

### Risk of bias

Apart from the studies by Burdett et al.^15^, Hesse et al.^23,24^, and Yamamoto et al.^31^, all included studies had a low risk of bias in the category of random sequence generation (Supplementary 2). Moreover, all included studies, except that by Pohl et al.^26^, had an unclear risk of bias in allocation concealment. In the section on blinding of participants and personnel, only Yamamoto et al. had a low risk of bias, and all others had a high risk of bias. All included studies were determined to have a high or unclear risk in the domain of blinding of outcome assessment. With respect to incomplete outcome data and selective reporting, the study by Gök et al.^22^ was the exception, and other included studies were assessed to have a low risk.

### Meta-analysis results

#### Walking speed (m/s)

**Figure 2:**
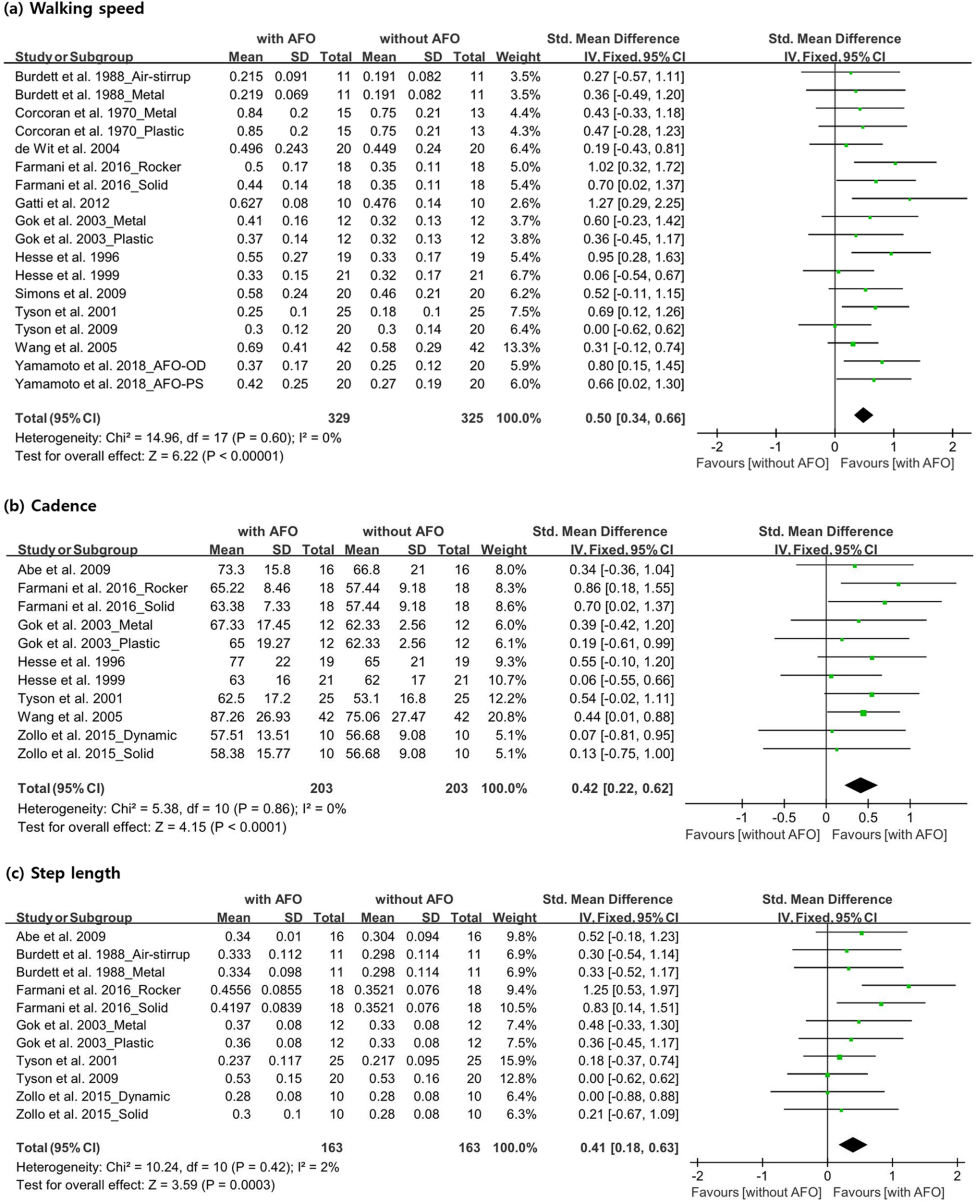
Forest plot showing the results of (**a**) walking speed, (**b**) cadence and (**c**) step length with or without ankle–foot orthosis use.

We measured the walking speed of 253 participants in 13 studies^15,17,18,20–24,27–31^. When the measurement units of the study data did not match, we standardized all data to meters per second. Eight studies^18,21,23,24,27–30^ evaluated walking speed in participants with and without AFO use. Each of the five studies^15,17,20,22,31^ evaluated two orthoses. Burdett et al.^13^ used air-stirrup and metal/plastic AFOs, and Corcoran et al.^17^ and Gök et al.^22^ evaluated plastic and metal AFOs. Farmani et al.^20^ used a solid AFO (SAFO) and a rocker bottom added to the solid AFO (RAFO). Yamamoto et al.^31^ used two types of metal AFOs: one was a plantarflexion stop (AFO-PS) with a Klenzak joint, and the other was a plantarflexion resistance (AFO-OD) with an oil damper. As the application of two AFOs was evaluated in five studies, the total number of participants mentioned above and that mentioned in Fig. 2 were inconsistent. A significant beneficial effect was observed (SMD, 0.50; 95% CI 0.34–0.66; P < 0.00001; I^2^, 0%) (Fig. 2).

#### Cadence (step/min)

Cadence data were extracted for 163 patients from eight studies^14,20,22–24,30,32^. As three of those studies^20,22,32^ used two AFOs, there were discrepancies in the total number of participants mentioned in Fig. 2. Farmani et al.^20^ evaluated a cohort wearing SAFOs and RAFOs, and Gök et al.^22^ evaluated hemiparetic patients using plastic and metal AFOs. Zollo et al.^32^ analyzed the application of SAFOs and dynamic AFOs. The other five studies^14,23,24,28,30^ evaluated cadence with and without AFO use. The cadence significantly increased when the participants wore AFOs (SMD, 0.42; 95% CI 0.22–0.62; P < 0.0001; I^2^, 0%) (Fig. 2).

#### Step length (m)

We evaluated the step length data in seven studies^14,15,20,22,28,29,32^ that included 112 participants in total. As four of these studies^15,20,22,32^ analyzed the application of two AFOs, the total number of participants shown in Fig. 2 is different. Burdett et al.^15^ performed step length measurements using air-stirrup and metal/plastic AFOs, and Farmani et al.^20^ measured the step lengths with SAFOs and RAFOs. Gök et al.^22^ used plastic and metal AFOs, and Zollo et al.^32^ used SAFOs and dynamic AFOs to measure the step length. Step length data that were reported in different units were standardized to values in meters. The results showed that with the application of AFOs, the step length significantly increased (SMD, 0.41; 95% CI 0.18–0.63; P = 0.0003; I^2^, 2%) (Fig. 2).

#### Stride length (m)

**Figure 3:**
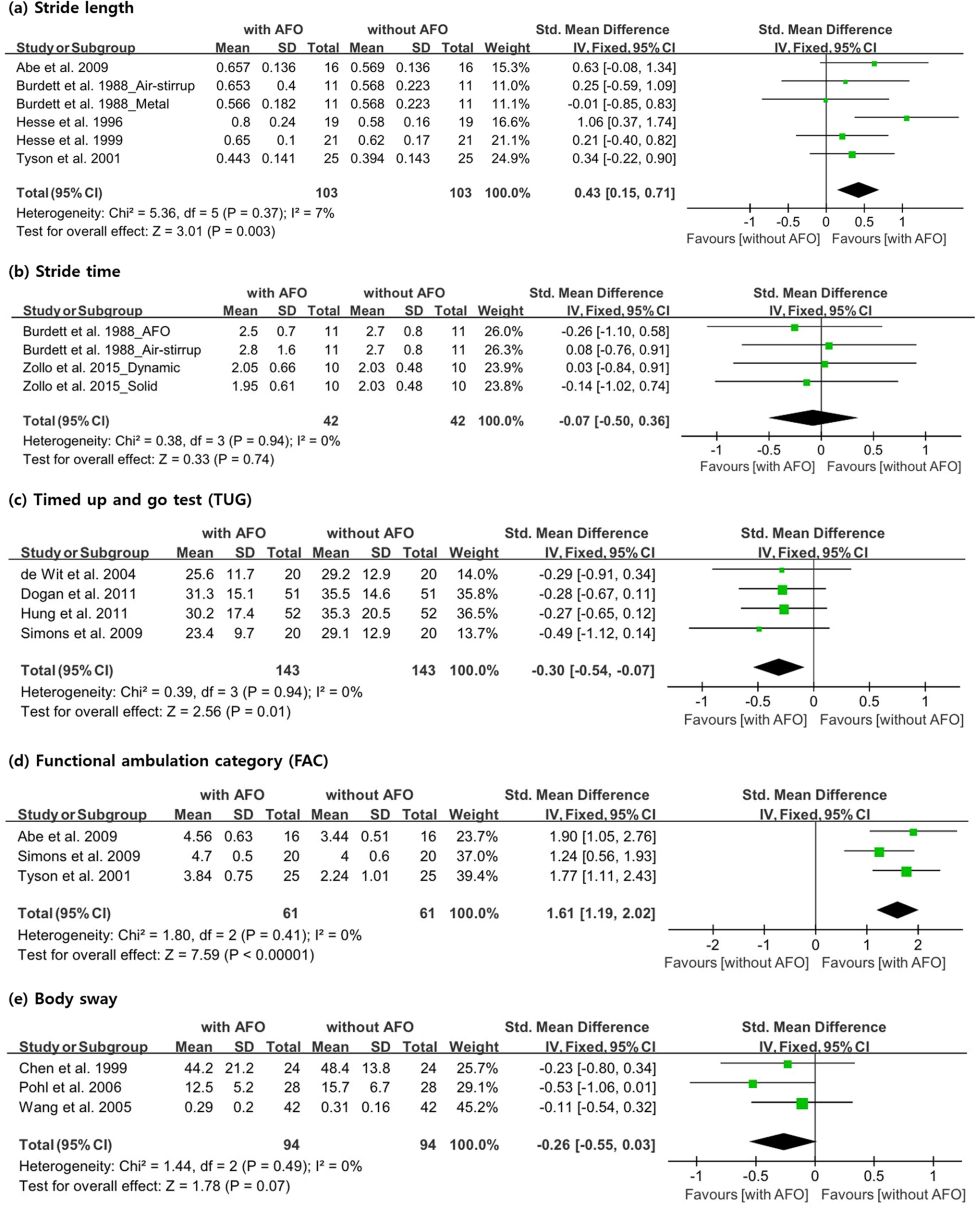
Forest plot showing the results of (**a**) stride length, (**b**) stride time, (**c**) timed up-and-go test, (**d**) functional ambulation category and (**e**) body sway with or without ankle–foot orthosis use.

Five studies^14,15,23,24,28^, with 92 participants, analyzed stride length. One of the studies^15^, in which two AFOs were used, had inconsistencies in the number of participants, as shown in Fig. 3. Four studies confirmed the stride length with and without AFO use, and Burdett et al.^15^ confirmed the effectiveness of using air-stirrup and metal/plastic AFOs. Data measured in different units were standardized to values in meters. AFO use had a significant beneficial effect in increasing the stride length (SMD, 0.43; 95% CI 0.15–0.71; P = 0.003; I^2^, 7%) (Fig. 3).

#### Stride time (s)

In two studies, the value of stride time was measured in 21 participants. As both studies used two AFOs, the number of participants was inconsistent with that calculated in Fig. 3. Burdett et al.^15^ used air-stirrup and metal/plastic AFOs, and Zollo et al.^32^ used SAFOs and dynamic AFOs. Stride time was longer when using air-stirrup and dynamic AFOs than when not using an AFO. Stride time was shorter when metal/plastic AFOs and SAFOs were used than when an AFO was not used. In the meta-analysis, no significant beneficial effect was observed (SMD, − 0.07; 95% CI − 0.50 to 0.36; P = 0.74; I^2^, 0%) (Fig. 3).

#### TUG

Four studies^18,19,25,27^, with 143 participants, conducted the TUG test. In all studies, the evaluation was performed by measuring the time it took for the participant to get up from the chair, walk 3 m, return to the chair, and sit down. All data were measured with and without AFO use, and TUG time significantly decreased when using an AFO (SMD, − 0.30; 95% CI − 0.54 to − 0.07; P = 0.01; I^2^, 0%) (Fig. 3).

#### FAC

In three studies, the mobility capabilities of 61 participants were evaluated using the FAC. Abe et al.^14^ and Simons et al.^27^ used the FAC in their study, which was categorized based on FAC scores ranging from 0 to 5 (requiring bilateral arm support for independent walking indoors and outdoors without supervision). Tyson et al.^28^ categorized the FAC into scores from 1 (continuous support) to 5 (independent anywhere). Strictly, the FAC score is a categorical variable, but for a meta-analysis, we treated it as a continuous variable. In a meta-analysis, the FAC score was significantly higher when using AFO than when not using it (SMD, 1.61; 95% CI 1.19–2.02; P < 0.00001; I^2^, 0%) (Fig. 3).

#### Body sway

Body sway was evaluated in 94 participants in three studies^16,26,30^, The evaluation tools used in each study were all different, but the key method of measurement was similar in all studies (i.e., body sway was measured while the participants were standing on a force platform with the eyes open). All three studies compared participants with and without AFO use, but the body sway did not change irrespective of the application of an AFO (SMD, − 0.26; 95% CI − 0.55 to − 0.03; P = 0.07; I^2^, 0%) (Fig. 3).

#### Ankle sagittal plane angle at initial contact (°)

**Figure 4:**
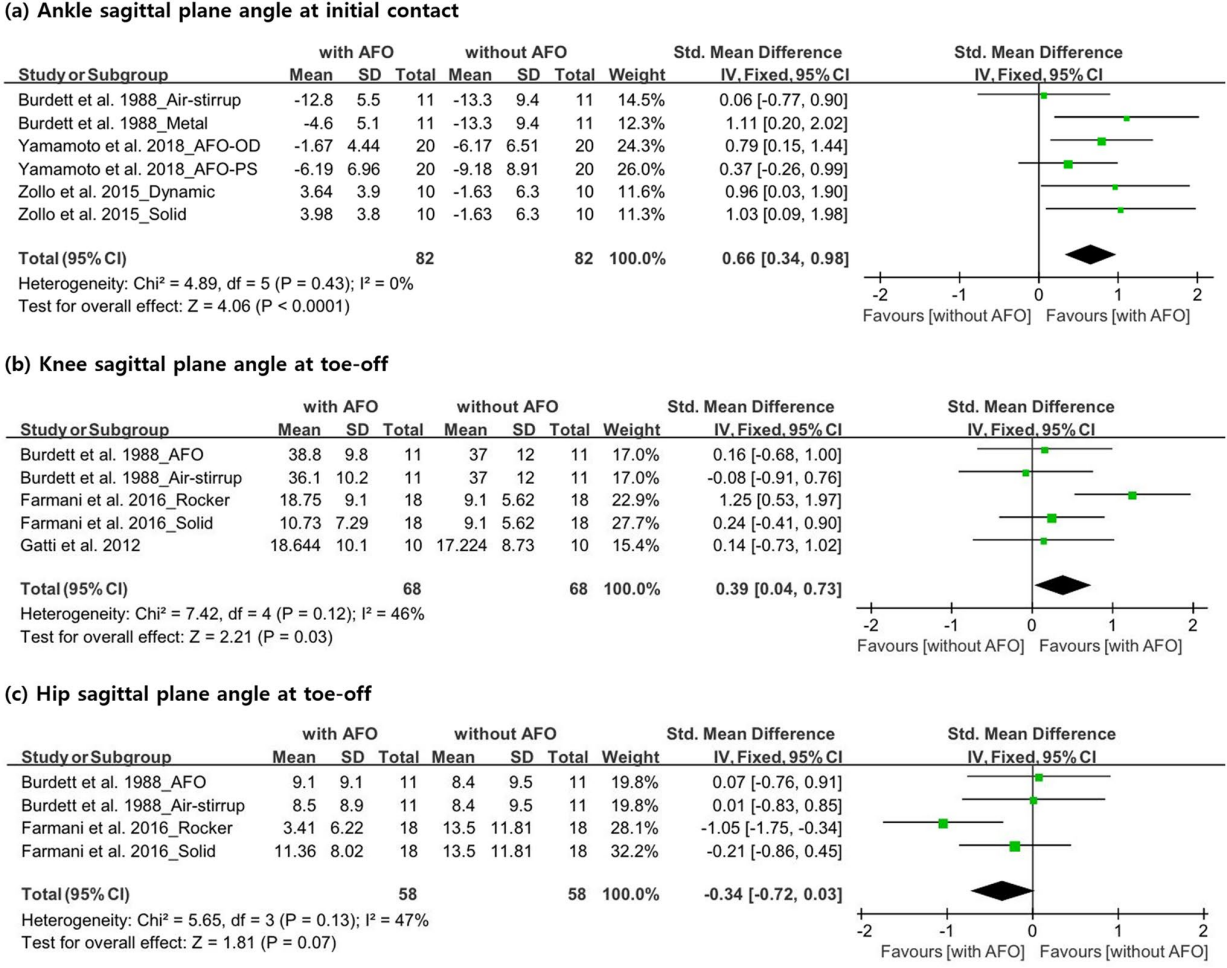
Forest plot showing the results of (**a**) ankle sagittal plane angle at initial contact, (**b**) knee sagittal plane angle at toe-off, and (**c**) hip sagittal plane angle at toe-off with or without ankle–foot orthosis use.

In three studies^15,31,32^, the sagittal plane angle of the ankle was measured at the initial contact in 41 participants. All studies included two AFOs; thus, the number of participants in these studies was inconsistent with the total number, as shown in Fig. 4. On the basis of the sagittal plane angle measurements, it was confirmed that an AFO can significantly assist dorsiflexion during the initial contact (SMD, 0.66; 95% CI 0.34–0.98; P < 0.0001; I^2^, 0%) (Fig. 4).

#### Knee sagittal plane angle at toe-off (°)

Three studies comprising 39 participants measured the sagittal plane angle of the knee at toe-off. As two of these studies^15,20^ used two AFOs, the total number of participants was different. Burdett et al.^15^ used air-stirrup and metal/plastic AFOs, and reported that a metal AFO was effective in improving knee flexion at toe-off. Farmani et al.^20^ used SAFOs and RAFOs and reported that both AFOs were effective. Gatti et al.^21^ used one AFO. Our meta-analysis confirmed that the angle of knee flexion increased when wearing an AFO (SMD, 0.39; 95% CI 0.04–0.73; P = 0.03; I^2^, 46%) (Fig. 4).

#### Hip sagittal plane angle at toe-off (°)

The data on hip sagittal plane angle at toe-off were analyzed in two studies that included 29 participants^15,20^. The number of participants was inconsistent between the real data and that in Fig. 4 because both studies used two AFOs. In one of these studies^15^, the hip extension angle was higher with an air-stirrup and metal/plastic AFO than without using an AFO. In contrast, Farmani et al.^20^ reported that the hip extension angle was lower when using an SAFO and a RAFO than when not using an AFO. In a meta-analysis, the hip sagittal plane angle at toe-off did not significantly change when wearing an AFO (SMD, − 0.34; 95% CI − 0.72 to 0.03; P = 0.07; I^2^, 47%) (Fig. 4).

### Publication bias

Publication bias was visually evaluated using a funnel plot showing the relationship between sample size and effect size, and statistically tested using Egger’s test, which tested for symmetry in the funnel plot. The graphic funnel plots of the changes in walking speed, cadence, step length, stride length, stride time, TUG, FAC, body sway, ankle sagittal plane angle at initial contact, and knee and hip sagittal plane angles at toe-off after wearing AFOs were symmetrical (Supplementary 3). Moreover, the P-value of Egger’s test was > 0.05, indicating an insignificant publication bias (walking speed = 0.1749, cadence = 0.4132, step length = 0.8847, stride length = 0.8547, stride time = 0.9386, TUG = 0.3001, FAC = 0.6786, body sway = 0.5562, ankle sagittal plane angle at initial contact = 0.3695, knee sagittal plane angle at toe-off = 0.4908, hip sagittal plane angle at toe-off = 0.4869).

## Discussion

In our meta-analysis, we evaluated the effectiveness of AFO use on improving gait function and balance in patients with stroke. We found that after wearing an AFO, the participants in the respective studies showed improvements in walking speed, cadence, step length, stride length, TUG test, and FAC score. Furthermore, the ankle sagittal plane angle at initial contact and knee sagittal plane angle at toe-off also increased. The I^2^ value, which is the ratio of the actual inter-study variance to the total variance, was analyzed to confirm the heterogeneity between the effect sizes of the studies included in the meta-analysis. Since the I^2^ value of all variables was less than 50, it is interpreted that the heterogeneity between the results of each analysis is small, and the average effect estimate was calculated using the fixed-effect model. However, the I^2^ values of variables except for knee and hip sagittal plane angle at toe-off were close to 0; conversely, both knee and hip sagittal plane angles at toe-off were close to 50, so it cannot be interpreted that the heterogeneity is very small. The funnel plot was used to visually evaluate the publication bias of the selected studies, and Egger's test was used as a statistical test to confirm whether the funnel plot was symmetrical. In the funnel plot, all variables were close to symmetric, and it was confirmed that there was no publication bias in Egger’s test results (p > 0.05). Therefore, in this meta-analysis, the risk that the actual effect may have been overestimated or that the actual effect may not be representative is considered low.

Many patients with stroke exhibit gait abnormalities, including slow speed, reduced step and stride length, and increased body sway^33,34^. Additionally, foot dragging due to weakened dorsiflexion of the ankle and extensor sparsity of the lower extremity may cause circumduction of the leg and steppage gait to compensate for the foot dragging^35,36^. In our study, we found that AFO use can increase gait function and balance (walking speed, cadence, step length, stride length, TUG, and FAC) and improve gait kinematic parameters (ankle sagittal plane angle at initial contact and knee sagittal plane angle at toe-off). We believe that an AFO improves gait function and corrects gait abnormalities by supporting dorsiflexion of the ankle and restricting plantarflexion and inversion. Previous studies have shown that the properties of AFOs that prevent knee hyperextension in the stance phase or correct ankle varus deformity in the stance/swing phase may affect the improvement of gait function^27^. Furthermore, the improvement in gait function can be due to the greater contribution of paralyzed lower extremities to weight-bearing or dynamic balance control^27^. An AFO prevents foot dragging along the ground, enhances mediolateral ankle stability in the stance phase by reducing step length, and promotes heel strike^20^. Increases in the angle of the ankle at initial contact and the knee angle at toe-off may indicate improved gait patterns. The improvement in knee flexion angle may be due to a decrease in premature gastrocnemius activity that occurs when wearing the AFO^21^. AFO is usually applied for patients with impairment of ankle dorsiflexion or hyper-plantarflexion in the swing phase. On the basis of the book on statistical power by Cohen^37^, AFO use has a large effect size on FAC; medium effect size on the ankle sagittal plane angle at initial contact; and small effect size on the walking speed, cadence, step length, stride length, and knee sagittal plane angle at toe-off. Generally, using AFO improves each gait function or the gait biomechanical parameters of patients with stroke to a small degree. All improvements in each gait component enhance the ambulation ability of patients with stroke, which is reflected in the large effect size of the improvement of FAC score after AFO wearing. This suggests that the overall gait function is enhanced as the biomechanical and kinematic parameters closely related to gait and balance are improved by wearing the AFO, which also affects the improvement of the FAC score.

In our meta-analysis, we found that AFO is useful for improving the gait function of patients with stroke. Specifically, AFO improves walking speed, cadence, step length, and stride length, particularly in patients with stroke with impairment of ankle dorsiflexion or hyper-plantarflexion in the swing phase. Moreover, it is considered beneficial in enhancing gait stability and general ambulatory ability. Additionally, AFO may be able to normalize gait patterns. However, this study had some limitations. First, the physical status, such as the degree of motor weakness, sensory deficits, and spasticity, of the included patients was not considered in each study. Second, the effects of rehabilitation or training other than the use of AFOs were not considered. Third, as the number of patients included in each study was small, our meta-analysis included a relatively small number of participants. Fourth, a limited number of databases were searched. Fifth, the study designs of the included studies were heterogeneous. Sixth, the study protocol was not registered or published in advance. In the future, a study should be conducted in which confounding variables such as the individual physical ability of the subjects and intervention in rehabilitation programs other than AFO are controlled, and more subjects should be included than in the current meta-analysis.

## Conclusion

Through this meta-analysis, we found that AFO was useful for improving gait speed, cadence, step length, and stride length in patients with stroke. In addition, since the sagittal plane angle of the ankle, knee, and hip is improved, patients with stroke with ankle dorsiflexor weakness or hyperplantarflexion problems can benefit by applying AFO. This meta-analysis provides basic data that can be used as a reference when providing AFO to patients with stroke in clinical practice.

## Supplementary Information

Supplementary Information 1.

Supplementary Information 2.

Supplementary Information 3.

## Data Availability

Some or all data, models, or code generated or used during the study are available from the corresponding author upon request.
