# Supplementary material for: Effectiveness of an ankle–foot orthosis on walking in patients with stroke: a systematic review and meta-analysis
Source: Sci Rep. 2021 Aug 5;11:15879. doi: 10.1038/s41598-021-95449-x (PMC8342539; doi:10.1038/s41598-021-95449-x)
Supplement: Supplementary file 1 — Supplementary Information 1. [file 41598_2021_95449_MOESM1_ESM.docx]

**Supplementary 1.** Search strategy.

**Supplementary 2.** Results of quality assessment of the selected studies.

**Supplementary 3.** Graphic funnel plots showing the differences in each assessment with and without ankle-foot orthosis use.
