# Supplementary material for: Effectiveness of an ankle–foot orthosis on walking in patients with stroke: a systematic review and meta-analysis
Source: Sci Rep. 2021 Aug 5;11:15879. doi: 10.1038/s41598-021-95449-x (PMC8342539; doi:10.1038/s41598-021-95449-x)

Title: Effectiveness of an Ankle-foot Orthosis on Walking and Balance in Patients with Stroke: A Systematic Review and Meta-analysis

Running Title: AFO for Patients with Stroke

Yoo Jin Choo[1] & Min Cheol Chang[2*]

[1]Production R&D Division Advanced Interdisciplinary Team, Medical Device Development Center, Daegu-Gyeongbuk Medical Innovation Foundation, Deagu, Republic of Korea

[2]Department of Rehabilitation Medicine, College of Medicine, Yeungnam University, Daegu, Republic of Korea

*Corresponding author: Min Cheol Chang, MD

Department of Physical Medicine and Rehabilitation, College of Medicine, Yeungnam University 317-1, Daemyungdong, Namku, Taegu 705-717, Republic of Korea

E-mail: wheel633@gmail.com

Funding: This study was supported by National Research Foundation of Korea Grant funded by the Korean government (Grant no. NRF-2021R1A2C1013073).

DISCLOSURE: The authors have no conflicts of interest to report.

Supplementary 1

Search strategy

A search strategy was developed to identify studies reporting the effectiveness of using an ankle-foot orthosis (AFO) in stroke patients.

The search keywords were combined as follows:

(stroke OR cerebrovascular disease)

AND

(orthotic devices OR braces OR splints OR foot OR ankle)

The search keywords were devised using a combination of subject indexing terms in the titles and abstracts. For the index related to stroke, "Search Methods for the Cochrane Stroke Group Specialized Register" was referenced, and the index related to orthosis is [O'Connor J, McCaughan D, McDaid C, et al. Health Technology Assessment, No. 20.55. Southampton (UK): NIHR Journals Library; 2016 Jul.] in "Appendix 1 Search strategies for the systematic review" was referenced.

Search strategy for: Search Methods for the Cochrane Stroke Group Specialized Register

**MEDLINE (Ovid) 1966 to June 2009 (week 2). Last searched 22 June 2009**
1. cerebrovascular disorders/
2. exp basal ganglia cerebrovascular disease/
3. exp brain ischemia/
4. exp carotid artery diseases/
5. stroke/ 6. exp brain infarction/
7. exp cerebrovascular trauma/
8. hypoxia-ischemia, brain/
9. exp intracranial arterial diseases/
10. exp intracranial arteriovenous malformations/
11. exp "Intracranial Embolism and Thrombosis"/
12. exp intracranial hemorrhages/
13. vasospasm, intracranial/
14. vertebral artery dissection/
15. aneurysm, ruptured/ and exp brain/
16. brain injuries/
17. brain injury, chronic/
18. exp carotid arteries/
19. endarterectomy, carotid/
20. *heart septal defects, atrial/ or foramen ovale, patent/
21. *atrial fibrillation/
22. (stroke or poststroke or post-stroke or cerebrovasc$ or brain vasc$ or cerebral vasc$ or cva$ or apoplex$ or isch?emi$ attack$ or tia$1 or neurologic$ deficit$ or SAH or AVM).tw.
23. ((brain$ or cerebr$ or cerebell$ or cortical or vertebrobasilar or hemispher$ or intracran$ or

intracerebral or infratentorial or supratentorial or MCA or anterior circulation or posterior circulation or basal ganglia) adj5 (isch?emi$ or infarct$ or thrombo$ or emboli$ or occlus$ or hypox$ or vasospasm or obstruction or vasculopathy)).tw.
24. ((lacunar or cortical) adj5 infarct$).tw.
25. ((brain$ or cerebr$ or cerebell$ or intracerebral or intracran$ or parenchymal or intraventricular or infratentorial or supratentorial or basal gangli$ or subarachnoid or putaminal or putamen or posterior fossa) adj5 (haemorrhage$ or hemorrhage$ or haematoma$ or hematoma$ or bleed$)).tw.
26. ((brain or cerebral or intracranial or communicating or giant or basilar or vertebral artery or berry or saccular or ruptured) adj5 aneurysm$).tw.
27. (vertebral artery dissection or cerebral art$ disease$).tw.
28. ((brain or intracranial or basal ganglia or lenticulostriate) adj5 (vascular adj5 (disease$ or disorder or accident or injur$ or trauma$ or insult or event))).tw.
29. ((isch?emic or apoplectic) adj5 (event or events or insult or attack$)).tw.
30. ((cerebral vein or cerebral venous or sinus or sagittal) adj5 thrombo$).tw.
31. (CVDST or CVT).tw.
32. ((intracranial or cerebral art$ or basilar art$ or vertebral art$ or vertebrobasilar or vertebral basilar) adj5 (stenosis or isch?emia or insufficiency or arterioscle ro$ or atheroscle ro$ or occlus$)).tw.
33. ((venous or arteriovenous or brain vasc$) adj5 malformation$).tw.
34. ((brain or cerebral) adj5 (angioma$ or hemangioma$ or haemangioma$)).tw.
35. carotid$.tw.
36. (patent foramen ovale or PFO).tw.
37. ((atrial or atrium or auricular) adj fibrillation).tw.
38. asymptomatic cervical bruit.tw.
39. exp aphasia/ or anomia/ or hemiplegia/ or hemianopsia/ or exp paresis/ or deglutition disorders/ or dysarthria/ or pseudobulbar palsy/ or muscle spasticity/
40. (aphasi$ or apraxi$ or dysphasi$ or dysphagi$ or deglutition disorder$ or swallow$ disorder$ or dysarthri$ or hemipleg$ or hemipar$ or paresis or paretic or hemianop$ or hemineglect or spasticity or anomi$ or dysnomi$ or acquired brain injur$ or hemiball$).tw.
41. ((unilateral or visual or hemispatial or attentional or spatial) adj5 neglect).tw.
42. or/1-41
43. Randomized Controlled Trials as Topic/
44. random allocation/
45. Controlled Clinical Trials as Topic/
46. control groups/
47. clinical trials as topic/ or clinical trials, phase i as topic/ or clinical trials, phase ii as topic/ or clinical trials, phase iii as topic/ or clinical trials, phase iv as topic/
48. Clinical Trials Data Monitoring Committees/
49. double-blind method/
50. single-blind method/
51. Placebos/
52. placebo effect/ 53. cross-over studies/
54. Multicenter Studies as Topic/
55. Therapies, Investigational/ 56. Drug Evaluation/
57. Research Design/
58. Program Evaluation/
59. evaluation studies as topic/
60. randomized controlled trial.pt.
61. controlled clinical trial.pt.

62. (clinical trial or clinical trial phase i or clinical trial phase ii or clinical trial phase iii or clinical trial phase iv).pt.
63. multicenter study.pt.
64. (evaluation studies or comparative study).pt.
65. meta analysis.pt.
66. meta-analysis as topic/ 67. random$.tw.
68. (controlled adj5 (trial$ or stud$)).tw.
69. (clinical$ adj5 trial$).tw.
70. ((control or treatment or experiment$ or intervention) adj5 (group$ or subject$ or patient$)).tw.
71. (surgical adj5 (group$ or subject$ or patient$)).tw.
72. (quasi-random$ or quasi random$ or pseudo-random$ or pseudo random$).tw.
73. ((multicenter or multicentre or therapeutic) adj5 (trial$ or stud$)).tw.
74. ((control or experiment$ or conservative) adj5 (treatment or therapy or procedure or manage$)).tw.
75. ((singl$ or doubl$ or tripl$ or trebl$) adj5 (blind$ or mask$)).tw.
76. (coin adj5 (flip or flipped or toss$)).tw.
77. latin square.tw.
78. versus.tw.
79. (cross-over or cross over or crossover).tw.
80. placebo$.tw.
81. sham.tw.
82. (assign$ or alternate or allocat$ or counterbalance$ or multiple baseline).tw.
83. controls.tw.
84. (treatment$ adj6 order).tw.
85. (meta-analy$ or metaanaly$ or meta analy$ or systematic review or systematic overview).tw.
86. or/43-85
87. 42 and 86
88. 87 not exp animals/
89. 87 and humans/
90. 88 or 89

EMBASE (Ovid) 1980 to June 2009 (week 25). Last searched 22 June 2009
1. Cerebrovascular Disease/
2. Basal Ganglion Hemorrhage/
3. cerebral artery disease/
4. Cerebrovascular Accident/
5. stroke/
6. stroke patient/ or stroke unit/
7. Vertebrobasilar Insufficiency/
8. exp Carotid Artery Disease/
9. exp Brain Hemangioma/
10. exp Brain Hematoma/
11. exp Brain Hemorrhage/
12. brain infarction/ or brain infarction size/ or brain stem infarction/ or cerebellum infarction/
13. exp Brain Ischemia/
14. exp Cerebrovascular Malformation/
15. exp intracranial aneurysm/
16. exp occlusive cerebrovascular disease/
17. brain injury/

18. brain stem injury/ or artery dissection/
19. cerebellum injury/
20. exp carotid artery/
21. exp carotid artery surgery/
22. carotid endarterectomy/
23. *heart atrium septum defect/ or heart foramen ovale/
24. *heart atrium fibrillation/
25. paradoxical embolism/
26. exp aphasia/ or hemiplegia/ or hemiparesis/ or paresis/ or spastic paresis/ or pseudobulbar palsy/ or hemianopia/ or homonymous hemianopia/ or dysphagia/ or dysarthria/ or dysphasia/ or spasticity/ or apraxia/ or dyspraxia/ or hemiballism/
27. (stroke or poststroke or post-stroke or cerebrovasc$ or brain vasc$ or cerebral vasc$ or cva$ or apoplex$ or isch?emi$ attack$ or tia$1 or neurologic$ deficit$ or SAH or AVM).tw.
28. ((brain$ or cerebr$ or cerebell$ or cortical or vertebrobasilar or hemispher$ or intracran$ or intracerebral or infratentorial or supratentorial or MCA or anterior circulation or posterior circulation or basal ganglia) adj5 (isch?emi$ or infarct$ or thrombo$ or emboli$ or occlus$ or hypox$ or vasospasm or obstruction or vasculopathy)).tw.
29. ((lacunar or cortical) adj5 infarct$).tw.
30. ((brain$ or cerebr$ or cerebell$ or intracerebral or intracran$ or parenchymal or intraventricular or infratentorial or supratentorial or basal gangli$ or subarachnoid or putaminal or putamen or posterior fossa) adj5 (haemorrhage$ or hemorrhage$ or haematoma$ or hematoma$ or bleed$)).tw.
31. ((brain or cerebral or intracranial or communicating or giant or basilar or vertebral artery or berry or saccular or ruptured) adj5 aneurysm$).tw.
32. (vertebral artery dissection or cerebral art$ disease$).tw.
33. ((brain or intracranial or basal ganglia or lenticulostriate) adj5 (vascular adj5 (disease$ or disorder or accident or injur$ or trauma$ or insult or event))).tw.
34. ((isch?emic or apoplectic) adj5 (event or events or insult or attack$)).tw.
35. ((cerebral vein or cerebral venous or sinus or sagittal) adj5 thrombo$).tw.
36. (CVDST or CVT).tw.
37. ((intracranial or cerebral art$ or basilar art$ or vertebral art$ or vertebrobasilar or vertebral basilar) adj5 (stenosis or isch?emia or insufficiency or arterioscleros$ or atheroscleros$ or occlus$)).tw.
38. ((venous or arteriovenous or brain vasc$) adj5 malformation$).tw.
39. ((brain or cerebral) adj5 (angioma$ or hemangioma$ or haemangioma$)).tw.
40. carotid$.hw.
41. (patent foramen ovale or PFO).tw.
42. ((atrial or atrium or auricular) adj fibrillation).tw.
43. asymptomatic cervical bruit.tw.
44. (aphasi$ or apraxi$ or dysphasi$ or dysphagi$ or deglutition disorder$ or swallow$ disorder$ or dysarthri$ or hemipleg$ or hemipar$ or paresis or paretic or hemianop$ or hemineglect or spasticity or anomi$ or dysnomi$ or
acquired brain injur$ or hemiball$).tw.
45. ((unilateral or visual or hemispatial or attentional or spatial) adj5 neglect).tw.
46. or/1-45
47. Randomized Controlled Trial/
48. Randomization/
49. Controlled Study/
50. control group/
51. clinical trial/ or phase 1 clinical trial/ or phase 2 clinical trial/ or phase 3 clinical trial/ or phase 4

clinical trial/ or controlled clinical trial/
52. Crossover Procedure/
53. Double Blind Procedure/
54. Single Blind Procedure/ or triple blind procedure/
55. latin square design/
56. Parallel Design/
57. placebo/
58. Multicenter Study/
59. experimental design/ or experimental study/ or quasi experimental study/
60. experimental therapy/
61. drug comparison/ or drug dose comparison/
62. drug screening/
63. EVALUATION/ or "EVALUATION AND FOLLOW UP"/ or evaluation research/ or clinical evaluation/
64. METHODOLOGY/
65. "types of study"/
66. research subject/
67. Comparative Study/
68. "systematic review"/
69. Meta Analysis/ 70. random$.tw.
71. (controlled adj5 (trial$ or stud$)).tw.
72. (clinical$ adj5 trial$).tw.
73. ((control or treatment or experiment$ or intervention) adj5 (group$ or subject$ or patient$)).tw.
74. (surgical adj5 (group$ or subject$ or patient$)).tw.
75. (quasi-random$ or quasi random$ or pseudo-random$ or pseudo random$).tw.
76. ((multicenter or multicentre or therapeutic) adj5 (trial$ or stud$)).tw.
77. ((control or experiment$ or conservative) adj5 (treatment or therapy or procedure or manage$)).tw.
78. ((singl$ or doubl$ or tripl$ or trebl$) adj5 (blind$ or mask$)).tw.
79. (coin adj5 (flip or flipped or toss$)).tw.
80. latin square.tw.
81. versus.tw.
82. (cross-over or cross over or crossover).tw.
83. placebo$.tw.
84. sham.tw.
85. (assign$ or alternate or allocat$ or counterbalance$ or multiple baseline).tw.
86. controls.tw.
87. (treatment$ adj6 order).tw.
88. (meta-analy$ or metaanaly$ or meta analy$ or systematic review or systematic overview).tw.
89. or/47-88
90. Human/
91. Nonhuman/
92. 90 and 91
93. 91 not 92
94. 89 not 93
95. 46 and 94

Search strategy for: Ovid MEDLINE® In-Process & Other Non-Indexed Citations and Ovid MEDLINE®

Date range searched: 1946 to 21 May 2014.

Date of search: 22 May 2014.

Search strategy

1. Orthotic Devices/ or Braces/ or Splints/ (16,320)
2. Gait/ (17,744)
3. Lower Extremity/ or Leg/ (61,929)
4. Hip/ or Hip Joint/ (28,943)
5. Knee/ or exp Knee Joint/ (51,355)
6. Ankle/ or Ankle Joint/ (16,707)
7. Foot/ or Foot Joints/ (20,388)
8. 1 and (2 or 3 or 4 or 5 or 6 or 7) (2732)
9. Foot Orthoses/ (145)
10. 8 or 9 (2870)
11. ((gait or "lower extremity" or "lower extremities" or "lower limb" or "lower limbs" or leg? or hip? or knee? or ankle? or foot or feet) adj3 (orthos* or orthot* or brace? or bracing or support)).ti,ab. (3590)
12. (heel adj2 (pad? or raise?)).ti,ab. (365)
13. ((shoe? and (modification? or insert? or "negative heel" or "negative heels")) or (rocker? or insole?)).ti,ab. (1507)
14. ((HKAFO? or KAFO? or SCKAFO? or AFO? or GRAFO? or RGO? or SWASH? or DAFO? or SAFO?) and (orthos* or orthot* or brace? or bracing)).ti,ab. (387)
15. (SMART? and walker).ti,ab. (10)
16. 11 or 12 or 13 or 14 or 15 (5269)
17. 10 or 16 (6735)
18. exp Knee Joint/ or Knee/ (51,355)
19. knee?.af. (11,4312)
20. 18 or 19 (115,529)
21. 17 and 20 (2085)

Selection of eligible studies

We searched trial registers using the "(stroke OR cerebrovascular disease) AND (orthotic devices OR braces OR splints OR foot OR ankle)" index, and databases including MEDLINE (Medical Literature Analysis and Retrieval System Online), CINAHL(Cumulative Index to Nursing and Allied Health Literature), Cochrane, Embase, and Scopus for studies published up to June, 2021. The results of the database searches were entered into an EndNote X9 library. Duplicates were deleted using the deduplication function in EndNote X9. Two reviewers preferentially removed irrelevant records for selection based on the titles and abstracts. Thereafter, the reviewers checked the full texts to finally select the papers that meet the selection criteria.

Each database was searched under the following conditions:

1. MEDLINE (537)

   - Article type: clical trial

   - Language: English

2. CINAHL (1,983)

   - None

3. Cochrane (1,596)

   - Article type: trial

4. Embase (245)

   - Diseases: cerebrovascular accident, brain ischemia, brain hemorrhage, cerebrovascular disease, brain infarction

   - Devices: orthosis, ankle foot orthosis, brace, splint, gait orthosis, walking aid

   - Study types: human, controlled study, major clinical study, clinical article, clinical trial

   - Publication types: article

5. Scopus (784)

   - Document type: article

   - Source type: journal

   - Language: English

Eligibility criteria

**Population**	Papers comparing the effectiveness of AFO use on gait in stroke patients with or without AFO use were included.
**Intervention**	Papers that evaluated the effectiveness of AFO use on gait were included. Papers that included more than one type of AFO were also included. However, papers on AFOs with electrical stimulation functions or robotic devices were excluded.
**Comparison**	Papers comparing the results of walking without an AFO with those of walking with an AFO were included.
**Outcome**	Studies were considered eligible for inclusion in this review if they reported on walking speed, cadence, step length, stride length, stride time, timed up-and-go time, functional ambulation category score, body sway, ankle sagittal plane angle at initial contact, knee sagittal plane angle at toe-off, and hip sagittal plane angle at toe-off
**Study design**	Clinical trials conducted with a experimental design, and prospective studies were included in this review. Studies published as abstracts, conference presentations, and case reports were excluded.
**Limitation**	To increase the reliability of the results, we included a study in which at least 10 subjects were recruited. Publications in a language other than English were excluded. Studies from all years were considered.

Supplementary 2

	Random sequence generation (selection bias)	Allocation concealment (selection bias)	Blinding of participants and personnel (performance bias)	Blinding of outcome assessment (detection bias)	Incomplete outcome data (attrition bias)	Selective reporting (reporting bias)	Other bias
Abe et al. 2009	+	?	−	−	+	+	+
Burdett et al. 1988_Air-stirrup	?	?	−	−	+	+	+
Burdett et al. 1988_Metal	?	?	−	−	+	+	+
Chen et al. 1999	+	?	−	−	+	+	+
Corcoran et al. 1970_Metal	+	?	−	−	+	+	+
Corcoran et al. 1970_Plastic	+	?	−	−	+	+	+
de Wit et al. 2004	+	?	−	−	+	+	+
Dogan et al. 2011	+	?	−	−	+	+	+
Farmani et al. 2016_Rocker	+	?	−	−	+	+	+
Farmani et al. 2016_Solid	+	?	−	−	+	+	+
Gatti et al. 2012	+	?	−	−	+	+	+
Gok et al. 2003_Metal	+	?	−	−	?	−	+
Gok et al. 2003_Plastic	+	?	−	−	?	−	+
Hesse et al. 1996	−	?	−	?	+	+	+
Hesse et al. 1999	−	?	−	−	+	+	+
Hung et al. 2011	+	?	−	−	+	+	+
Pohl et al. 2006	+	+	−	−	+	+	+
Simons et al. 2009	+	?	−	−	+	+	+
Tyson et al. 2001	+	?	−	−	+	+	+
Tyson et al. 2009	+	?	−	−	+	+	+
Wang et al. 2005	+	?	−	−	+	+	+
Yamamoto et al. 2018_AFO-OD	−	?	+	?	+	+	+
Yamamoto et al. 2018_AFO-PS	−	?	+	?	+	+	+
Zollo et al. 2015_Dynamic	+	?	−	−	+	+	+
Zollo et al. 2015_Solid	+	?	−	−	+	+	+

Supplementary 3

(a) Walking speed

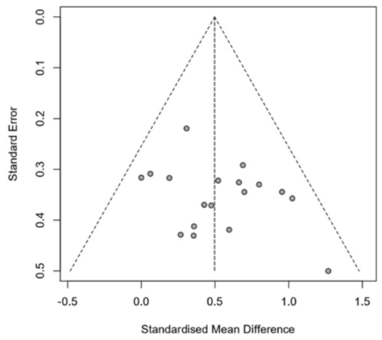

(b) Cadence

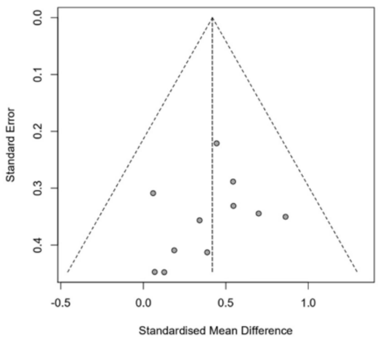

(c) Step length

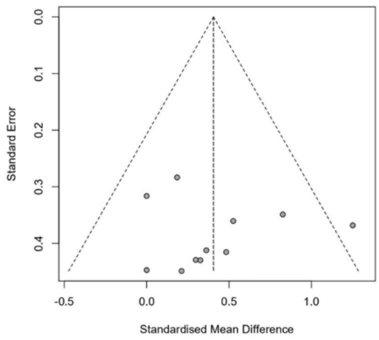

(d) Stride length

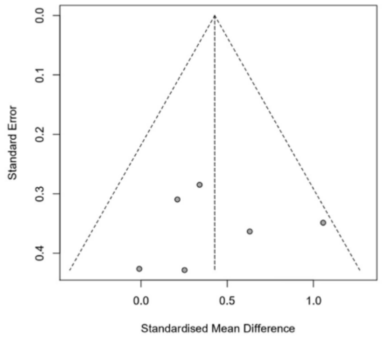

(e) Stride time

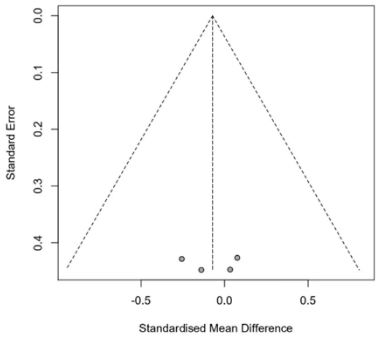

(f) TUG

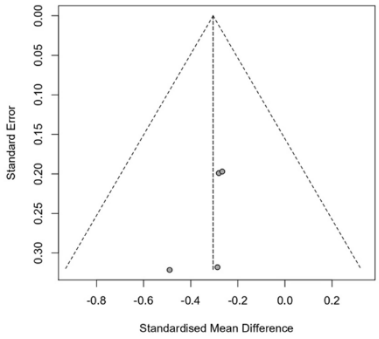

(g) FAC

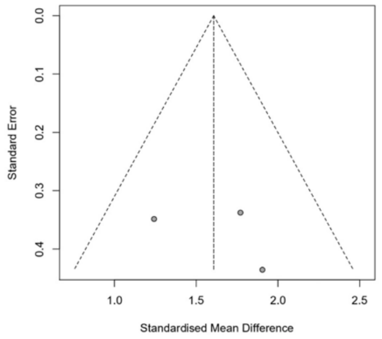

(h) Body sway

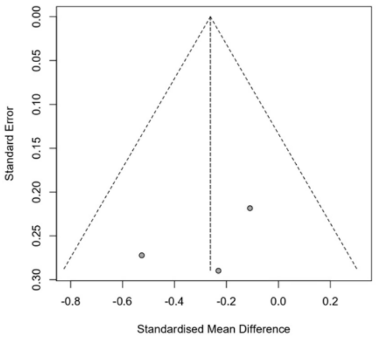

(i) Ankle angle

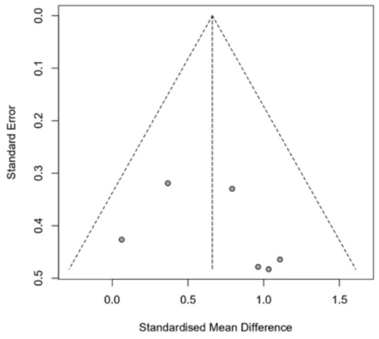

(j) Knee angle

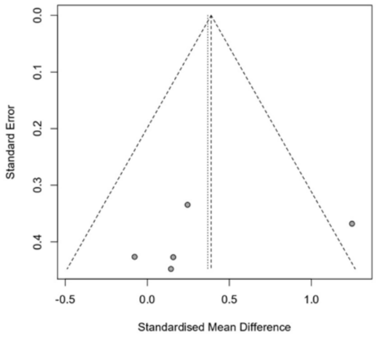

(k) Hip angle

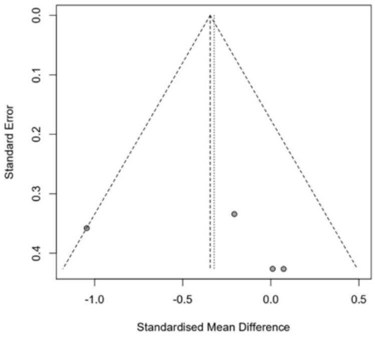

Supplement: Supplementary file 2 — Supplementary Information 2. [file 41598_2021_95449_MOESM2_ESM.pdf]
